# Pharmacological Management of Obesity in Patients with Polycystic Ovary Syndrome

**DOI:** 10.3390/biomedicines11020496

**Published:** 2023-02-08

**Authors:** Christodoula Kourtidou, Konstantinos Tziomalos

**Affiliations:** First Propedeutic Department of Internal Medicine, Medical School, Aristotle University of Thessaloniki, AHEPA Hospital, 54636 Thessaloniki, Greece

**Keywords:** polycystic ovary syndrome, obesity, overweight, glucagon-like peptide-1 receptor agonists, dipeptidyl peptidase-4 inhibitors, sodium glucose contrasporter-2 inhibitors, phosphodiesterase-4 inhibitors

## Abstract

Polycystic ovary syndrome (PCOS) is a common endocrine disorder in women of reproductive age. A substantial proportion of patients with PCOS are either overweight or obese, and excess body weight aggravates the hormonal, reproductive and metabolic manifestations of PCOS. In recent years, several studies evaluated the role of various pharmacological agents in the management of obesity in this population. Most reports assessed glucagon-like peptide-1 receptor agonists and showed a substantial reduction in body weight. More limited data suggest that sodium-glucose cotransporter-2 inhibitors and phosphodiesterase-4 inhibitors might also be effective in the management of obesity in these patients. In the present review, we discuss the current evidence on the safety and efficacy of these agents in overweight and obese patients with PCOS.

## 1. Introduction

The prevalence of obesity has rapidly increased over the past 25 years worldwide [1]. Obesity is associated with an increased risk for cardiovascular disease (CVD), type 2 diabetes mellitus (T2DM), cancer, chronic kidney disease, musculoskeletal disorders and reduced life expectancy [1,2]. On the other hand, polycystic ovary syndrome (PCOS) represents the commonest endocrine disorder among women of reproductive age [3,4]. The prevalence of PCOS varies depending on the diagnostic criteria applied; according to the diagnostic criteria of the National Institutes of Health, Rotterdam, and Androgen Excess and PCOS Society, the prevalence of PCOS is approximately 6%, 10% and 15%, respectively [5]. The components of PCOS are androgen excess, polycystic ovarian morphology and dysfunction in ovulation [6]. PCOS is linked to various metabolic disorders, including obesity, insulin resistance, T2DM and CVD [7,8,9,10].

Approximately 50–80% of patients with PCOS are obese [6,11]. In addition, women with PCOS predominantly have visceral adiposity [12]. Obesity affects fertility [13] and is associated with a worse metabolic profile in patients with PCOS [14]. Several epidemiologic studies suggested that weight loss and adipose fat loss improve fertility and ovulation rates in obese women with PCOS [15,16,17]. Indeed, a 5–10% weight loss is sufficient to ameliorate the metabolic and reproductive abnormalities in patients with PCOS [18].

Lifestyle changes represent the cornerstone of the management of obesity in patients with PCOS [18]. However, weight loss is frequently suboptimal, even in patients who adhere to lifestyle recommendations [18]. Moreover, weight regain often occurs in patients who originally lose weight [18]. Accordingly, several drugs, including orlistat, metformin and sibutramine, have been evaluated in the past for the management of obesity in PCOS [19,20]. However, these agents appear to have limited efficacy and/or unfavorable safety profile [19,20]. The aim of the present review is to summarize the evidence regarding the role of emerging pharmacologic agents for the treatment of obesity in patients with PCOS.

## 2. Glucagon-Like Peptide-1 (GLP-1) Receptor Agonists

GLP-1 receptor agonists enhance glucose-dependent insulin secretion and inhibit glucagon release [21]. GLP-1 receptor agonists are currently indicated for the treatment of T2DM and obesity [22]. GLP-1 receptor agonists induce weight loss in patients with or without T2DM [23,24,25,26]. The effect of GLP-1 receptor agonists on weight control could be partly explained by the altered appetite signaling and the prolonged gastric emptying [27,28] mediated by several neural pathways [29,30]. Several preclinical studies evaluated the potential mechanisms underpinning the effect of GLP-1 receptor agonists on body weight and adiposity [31,32,33,34]. These studies showed the potential relationship between GLP-1 agonism and inhibition of fatty acid synthase expression [31], brown adipose tissue thermogenesis mediated by AMP-activated protein kinase (AMPK) and sirtuin 1 (SIRT-1) [32,34] and lipolysis and fatty acid oxidation mediated by SIRT-1 [33].

A number of studies evaluated the effect of GLP-1 agonists on weight in patients with PCOS (Table 1). Most of these studies assessed liraglutide at the dose of 1.2–1.8 mg/day, i.e., the dose recommended for the management of T2DM. In a double-blind trial, 65 overweight women with PCOS were randomized to receive liraglutide 1.8 mg/day or a placebo for 26 weeks [35]. Treatment with liraglutide resulted in a mean reduction in body weight of 5.2 kg, body mass index (BMI) of 1.8 kg/m^2^, total body fat mass of 2.8 kg and visceral adipose tissue (VAT) of 21.9 cm^3^, more than placebo [35]. In a 12-week, open-label, randomized study of 28 obese women with a new diagnosis of PCOS, liraglutide 1.2 mg/day and metformin 1000 bid induced similar reductions in weight (−2.52 kg), BMI (−0.98 kg/m^2^), waist circumference (−3.38 cm) and total body fat mass (−1.26%) [36].

A number of studies evaluated liraglutide in patients with PCOS who had inadequate weight loss response to metformin before the intervention (<5 kg or <5% weight loss). In an open-label, prospective study of 36 obese women with PCOS, patients were randomly assigned to metformin 1000 mg bid or liraglutide 1.2 mg/day or the combination of metformin 1000 mg bid and liraglutide 1.2 mg/day [37]. After 12 weeks, 22% and 16% of the patients in the combination treatment and liraglutide monotherapy group lost ≥5% body weight compared with none in the metformin monotherapy group [37]. In an observational study of 84 overweight/obese women with PCOS, treatment with liraglutide 1.2–1.8 mg/day for a mean period of 27.8 weeks induced a reduction in weight of 9.0 kg and a reduction in BMI of 3.2 kg/m^2^ [38]. In another study of 36 obese patients with PCOS, a transition from metformin to liraglutide 1.2 mg/day for 12 weeks reduced weight by 3.8 kg [39].

**Table 1:** Major studies evaluating the effects of glucagon-like peptide-1 (GLP-1) receptor agonists on obesity in patients with polycystic ovary syndrome.

Reference	GLP-1 Agonist	n	Follow-Up (Weeks)	Baseline Body Mass Index (kg/m^2^)	Lifestyle Intervention	Metformin Treatment	Main Results
[35]	Liraglutide 1.2–1.8 mg/day	65	26	33.3	No	No	Greater reduction in body weight than placebo
[36]	Liraglutide 1.2 mg/day	28	12	39.5	No	Comparator arm	Similar weight loss with metformin
[40]	Liraglutide 3.0 mg/day	67	32	42.7	Diet and exercise	No	57% experienced ≥5% weight loss compared with 22% in the placebo group
[41]	Liraglutide 3.0 mg/day	28	12	38.3	Diet and exercise	Comparator arm	Greater reduction in body mass index and waist circumference and waist circumference than metformin 1000 mg bid and liraglutide 1.2 mg/day

Few studies evaluated the effects of liraglutide 3.0 mg/day, i.e., the dose indicated for the management of obesity. In a 32-week, randomized, double-blind, placebo-controlled trial of 67 obese women with PCOS, 57% of patients treated with liraglutide 3 mg/day experienced ≥5% weight loss compared with 22% in the placebo group [40]. Patients treated with liraglutide also experienced greater reductions in BMI (2.5 vs. 0.5 kg/m^2^), waist circumference (10 vs. 6 cm) and total body % fat (1.6 vs. 0.3%) compared with placebo. In a 12-week, randomized, open-label study of 28 obese patients with PCOS, the combination of metformin 1000 mg bid and liraglutide 1.2 mg/day induced similar weight loss with liraglutide 3 mg/day (3.6 vs. 6.3 kg, respectively) but the latter was associated with a greater reduction in BMI (1.3 vs. 2.2 kg/m^2^, respectively) and waist circumference (2.2 vs. 4.2 cm, respectively) [41]. In a meta-analysis of three randomized controlled studies, liraglutide reduced body weight more than metformin [42].

Several older studies evaluated another GLP-1 receptor agonist, exenatide, in overweight or obese patients with PCOS. In an open-label study of 20 overweight/obese patients with PCOS, exenatide 5 μg bid for 4 weeks and 10 μg bid for another 12 weeks reduced weight by 3%, reduced BMI by 1.2 kg/m^2^ and hip circumference by 3.0 cm but had no effect on waist circumference [43]. Compared with metformin, in a 24-week, open-label, randomized study of 158 overweight/obese patients with PCOS, exenatide 10 μg bid reduced weight and total fat % by 2.0 kg and 3.56% more than metformin 1000 mg bid, respectively [44]. In another randomized, comparative study of 63 overweight/obese patients with PCOS, exenatide 10 μg bid for 12 weeks resulted in a greater reduction in weight (by 1.7 kg), BMI (by 0.7 kg/m^2^) and abdominal girth (by 3.0 cm) compared with metformin 1000 mg bid [45]. Finally, some studies evaluated the combination of exenatide with metformin compared with monotherapy. In an open-label, randomized study of 40 overweight/obese patients with PCOS, the combination of metformin 500 mg bid and exenatide 2 mg/day reduced weight, BMI and waist circumference more than metformin monotherapy (by 1.7 kg, 0.7 kg/m^2^ and 2.9 cm more, respectively) [46]. In a 24-week, open-label, randomized study of 42 overweight patients with PCOS, monotherapy with exenatide 10 μg bid and combination therapy with exenatide 10 μg bid and metformin 1000 mg bid resulted in greater weight loss (by 1.6 and 4.0 kg more, respectively) than monotherapy with metformin 1000 mg bid [47]. Abdominal girth decreased in the combination group at 24 weeks, whereas it increased in the metformin monotherapy group (by 6.0 and 0.5 cm, respectively) [47].

Another GLP-1 receptor agonist, semaglutide, is approved for the management of obesity and appears to be the most effective member of this class in terms of weight loss [48]. However, in a recent single-blind study of 20 obese patients with PCOS, semaglutide 1.0 mg once weekly administered for 12 weeks induced similar weight loss with a placebo (5 and 2 kg, respectively) [49]. Tirzepatide, a dual GLP-1 and glucose-dependent insulinotropic peptide (GIP) receptor agonist appears to induce even greater weight loss than semaglutide [48] but has not been evaluated in patients with PCOS.

GLP-1 receptor agonists were generally well-tolerated in these studies, and the most frequent adverse events were nausea and constipation [35,40].

## 3. Dipeptidyl Peptidase-4 (DPP-4) Inhibitors

By blocking the degradation of GLP-1, DPP-4 inhibitors stimulate insulin secretion from pancreatic β-cells and inhibit glucagon secretion from pancreatic α-cells [50,51]. DPP-4 inhibitors are indicated for the treatment of T2DM [50,51]. In animal models, the DPP-4 inhibitor teneligliptin improved brown adipose tissue function, reduced body weight and fat mass and increased energy expenditure [51,52,53]. Furthermore, in mice, weight loss by DPP-4 inhibition was attributed to decreased food intake [54], greater energy expenditure and changes in the metabolic function of white adipose tissue [55]. Furthermore, DPP-4 inhibition was associated with improved energy expenditure and reduced adiposity mediated by GLP-1 and melanocortin-4 pathways [56]. However, in a rat model of PCOS, sitagliptin did not affect body weight [57].

Few studies evaluated the effects of DPP-4 inhibitors in patients with PCOS (Table 2). In a double-blind, randomized, placebo-controlled study of 18 overweight patients with PCOS, sitagliptin 100 mg/day for 1 month reduced VAT mass by 87.0 g and volume by 92.2 cm^3^ more than placebo [58]. In a randomized, open-label study of 28 obese patients with PCOS who had discontinued metformin, sitagliptin 100 mg/day combined with lifestyle intervention for 12 weeks, maintained body weight, waist circumference and VAT mass compared with an increase in these parameters with lifestyle intervention alone [59]. In a 12-week, randomized, open-label study of 24 obese patients with PCOS previously treated with liraglutide, sitagliptin 100 mg/day combined with metformin 1000 mg bid resulted in no change in weight, whereas monotherapy with metformin 1000 mg bid was associated with a weight gain of 4.7 kg [60]. In a randomized, single-blind study of 34 patients with PCOS, metformin 2000 mg/day, saxagliptin 5 mg, and the combination of saxagliptin 5 mg and metformin 2000 mg/day for 16 weeks induced similar reductions in BMI and waist circumference (by 0.8 kg/m^2^ and 2.5 cm, respectively) [61]. In these studies, the incidence of adverse events did not differ between patients who were treated with DPP-4 inhibitors and those who received a placebo or lifestyle intervention alone [58,59].

## 4. Sodium Glucose Contrasporter-2 (SGLT-2) Inhibitors

SGLT-2 inhibitors increase urinary glucose excretion by inhibiting renal glucose reabsorption and, in consequence, reduce hyperglycemia [62,63]. Apart from their role in the management of glycemia, SGLT-2 inhibitors facilitate weight loss [64,65]. The weight loss effect of SGLT-2 inhibitors was preserved in studies with follow-ups up to 4 years [66,67]. Several studies showed that in patients treated with SGLT-2 inhibitors, weight loss is accompanied by total body fat mass and VAT volume reduction [66,68]. SGLT-2 inhibitors enhance fat utilization and energy expenditure by the browning of fat [69,70]. In preclinical studies, canaglifozin and dapaglifozin increased sympathetic innervation [71,72] and enhanced mitochondrial function and fatty acid oxidation in adipose tissue [73]. Canaglifozin may enhance energy metabolism by activating the AMPK and SIRT-1 pathways [74] and activate lipolysis and fat mass reduction mediated by FGF21-related mechanisms [75]. Empagliflozin also reduced fat mass in a rat model [76].

In a 12-week, randomized, open-label study of 39 overweight or obese patients with PCOS, empagliflozin 25 mg/day reduced body weight, BMI, waist circumference, hip circumference and total body fat mass more than metformin 1500 mg/day (by 0.2%, 0.3%, 1.4%, 0.9% and 2.5%, respectively) (Table 3) [77]. In an open-label, randomized trial of 41 overweight/obese patients with PCOS, canaglifozin 100 mg/day combined with metformin 1000 mg bid for 3 months induced similar reductions in weight and BMI compared with metformin 1000 mg bid monotherapy (by 6.2 kg and 2.35 kg/m^2^, respectively) [78]. In a randomized, placebo-controlled, double-blind trial of 20 patients with PCOS, licogliflozin 50 mg tid, a dual SGLT1/2 inhibitor, did not induce weight loss after 2 weeks [79]. In a 24-week, randomized, single-blind study of 92 obese patients with PCOS, the combination of exenatide 2 mg weekly and dapaglifozin 10 mg/day induced similar body weight with phentermine/topiramate 7.5/46 mg per day and greater than exenatide or dapagliflozin monotherapy or dapaglifozin combined with metformin 2000 mg/day (by 6.0, 9.0, 4.1, 1.4 and 1.8 kg, respectively) [80]. The reduction in total body fat was also similar with exenatide/dapagliflozin combination and phentermine/topiramate and greater than exenatide or dapagliflozin monotherapy or dapaglifozin/metformin combination (by 1.8, 2.2, 0.8, 0.6 and 0.0%, respectively) [80]. Major studies that evaluated the effects of SGLT-2 inhibitors on obesity in patients with polycystic ovary syndrome are shown in Table 3. In these studies, the commonest adverse event in patients who received SGLT-2 inhibitors was genitourinary system infections [80].

## 5. Phosphodiesterase-4 (PDE-4) Inhibitors

PDE-4 inhibitors attenuate cAMP metabolism and its further signaling [81]. PDE-4 inhibitors have been evaluated for the treatment of several diseases, including chronic obstructive pulmonary disease [82] and metabolic disorders such as T2DM, obesity and hypertension [83]. Roflumilast, a PDE-4 inhibitor, has been associated with weight loss [84,85]. In overweight/obese patients, roflumilast was associated with reduced energy intake and decreased fat mass [86]. In mice, roflumilast prevented visceral fat and weight gain and reduced adipogenesis and lipolysis mediated by the AMPK pathway [87]. Other studies showed that PDE-4 inhibition lowers body weight by enhancing energy expenditure and lipolysis [88,89,90].

Only two studies evaluated the effects of PDE-4 inhibitors on obesity in patients with polycystic ovary syndrome (Table 4). In a 12-week, randomized, open-label study of 31 obese patients with PCOS, the combination of roflumilast 500 μg/day and metformin 1000 mg bid reduced body weight, BMI and VAT area more than metformin (by 3.3 kg, 2.5 kg/m^2^ and 36.9 cm^2^, respectively) [91]. In another 12-week, randomized, open-label study of 41 obese patients with PCOS, roflumilast 500 μg/day reduced body weight to a similar degree as liraglutide 1.2 mg/day and more than metformin 1000 mg bid (by 2.1, 3.1 and 0.2 kg, respectively) [92]. Nausea, diarrhea and headache were the most frequent adverse events in patients treated with roflumilast [92].

## 6. Conclusions

Several studies evaluated the safety and efficacy of various pharmacological agents in the management of obesity in patients with PCOS. However, most of these studies were small and had short follow-ups. Available evidence suggests that GLP-1 receptor agonists, particularly liraglutide, might represent the pharmacotherapy of choice in overweight/obese patients with PCOS. An important advantage of liraglutide is that it is licensed for the management of obesity. Other agents, including SGLT-2 inhibitors and PDE-4 inhibitors, also appear to be useful but are less well-studied in this population and are not yet licensed for weight loss. Even though GLP-1 receptor agonists are more expensive than DPP-4, SGLT-2 and PDE-4 inhibitors, they appear to be more cost-effective than the latter because they also induce considerably greater weight loss. Regarding the duration of treatment, it is possible that life-long therapy will be required to prevent weight regain. However, all these agents are contraindicated during pregnancy, and therefore appropriate contraceptive methods should be followed during treatment with these drugs. More studies are needed to evaluate whether combination therapy might be more effective than monotherapy in the management of obesity in PCOS. Moreover, all the studies that evaluated these agents in patients with PCOS had a short duration, and none assessed the effects of these treatments on cardiovascular morbidity. Therefore, larger studies with long follow-ups are needed to firmly establish the safety and efficacy of these therapies in patients with PCOS.

## Figures and Tables

**Table 2:** Major studies evaluating the effects of dipeptidyl peptidase-4 (DPP-4) inhibitors on obesity in patients with polycystic ovary syndrome.

Reference	DPP-4 Inhibitor	n	Follow-Up (Weeks)	Baseline Body Mass Index (kg/m^2^)	Lifestyle Intervention	Metformin Treatment	Main Results
[55]	Sitagliptin 100 mg/day	18	4	34.1	No	No	Greater reduction in visceral adipose tissue than placebo
[56]	Sitagliptin 100 mg/day	28	12	36.9	Diet and exercise	Prior	Stable body weight compared with an increase in body weight with lifestyle intervention
[58]	Saxagliptin 5 mg/day	34	16	41.0	No	Comparator arm	Similar reduction in body mass index and waist circumference with metformin and metformin/saxagliptin

**Table 3:** Major studies evaluating the effects of sodium-glucose cotransporter-2 (SGLT-2) inhibitors on obesity in patients with polycystic ovary syndrome.

Reference	SGLT-2 Inhibitor	n	Follow-Up (Weeks)	Baseline Body Mass Index (kg/m^2^)	Lifestyle Intervention	Metformin Treatment	Main Results
[74]	Empagliflozin 25 mg/day	39	12	37.9	No	Comparator arm	Greater reduction in body weight than metformin
[75]	Canagliflozin 100 mg/day	41	12	30.2	No	Comparator arm	Canagliflozin/metformin induced similar reductions in body weight with metformin
[77]	Dapagliflozin 10 mg/day	92	24	38.5	Diet and exercise	Comparator arm	Dapagliflozin/exenatide induced similar body weight loss with phentermine/topiramate and greater than exenatide, dapagliflozin and dapaglifozin/metformin

**Table 4:** Major studies evaluating the effects of phosphodiesterase-4 (PDE-4) inhibitors on obesity in patients with polycystic ovary syndrome.

Reference	PDE-4 Inhibitor	n	Follow-Up (Weeks)	Baseline Body Mass Index (kg/m^2^)	Lifestyle Intervention	Metformin Treatment	Main Results
[88]	Roflumilast 500 μg/day	31	12	36.4	No	Comparator arm	Roflumilast/metformin reduced body weight more than metformin
[89]	Roflumilast 500 μg/day	41	12	38.6	No	Comparator arm	Similar reduction in body weight compared with liraglutide and greater than with metformin

## Data Availability

Not applicable.
